# Assessing the carbon footprint of a Colombian University Campus using the UNE-ISO 14064–1 and WRI/WBCSD GHG Protocol Corporate Standard

**DOI:** 10.1007/s11356-022-22119-4

**Published:** 2022-08-13

**Authors:** Natalia Cano, Linda Berrio, Elizabeth Carvajal, Santiago Arango

**Affiliations:** 1 Facultad de Minas, Departamento de Geociencias Y Medio Ambiente, Universidad Nacional de Colombia – Sede Medellín, Cra 80 No 65-223, M2-301 Bloque, Colombia; 2 Department Green Chemistry and Technology, Faculty of Bioscience Engineering, Ghent University, Gent, Belgium; 3 Facultad de Minas, Departamento de Ciencias de La Decisión Y La Computación, Universidad Nacional de Colombia – Sede Medellín, Cra 80 No 65-223, M8 Bloque, Colombia

**Keywords:** Higher education institutions, Greenhouse gas emissions, Carbon footprint, Mitigation, Reduction

## Abstract

Higher education institutions (HEIs) transfer skills and knowledge between industries, the government, and the public, playing a vital role at educating future leaders in creating a globally sustainable system. Therein, evaluating greenhouse gas emissions from an educational institute is the first step towards the proposed reduction targets at the local, national, and international levels. In this research, we report the first approximate carbon footprint calculation of emissions corresponding to scope 1, scope 2, and scope 3 emissions for the main urban campuses of Universidad Nacional de Colombia, Medellín, using the UNE-ISO 14064–1 and WRI/WBCSD GHG Protocol Corporate standard. The carbon footprint in 2019 was approximately 7250.52 tons CO_2 eq_, and 0.432 tons CO_2 eq_ per person. Scope 1 emissions accounted for about 2.84% of the carbon footprint, while scope 2 and 3 emissions each contributed nearly 14% and 83%, respectively. The largest sources of greenhouse gas emissions were the transportation process (58.51%), the wastewater process (17.01%), followed by electricity consumption (14.03%), and the e-mails that are sent (6.51%). It is suggested some proposals and strategies for mitigating and reducing emissions. Colombian HEIs exhibit the lowest ton of CO_2 eq_. per person compared to the other HEIs. Several reasons explain this behavior across the document such as geographic location (climate and topography), cultural factors (consumption patterns and types of transportation), population size, typology (public or private), gross domestic product (GDP) of each country, and methodology implemented. Results cannot be extrapolated to the Colombian case for the differences in the local conditions; therefore, it is not possible to get solid conclusions on the CF behavior in the Colombian HEIs. In this research, we provide for the first time a carbon footprint calculation where the sociological, political, and geographic conditions not extrapolated representing a valuable contribution to the HEI’s of the country. This research can be a benchmark in the carbon footprint calculation and a methodological contribution to HEIs in the country.

## Introduction

Climate change is one of the most serious global environmental problems caused by anthropogenic actions (Lima et al. 2020). It has a significant negative impact on natural resources, terrestrial and aquatic ecosystems, human health, and human systems (García-Leoz et al. 2018; Guemene Dountio et al. 2016). Anthropogenic greenhouse gas (GHG) emissions are a consequence of human activities and play an important role in climate change (Clabeaux et al. 2020; Ridhosari and Rahman 2020). Therefore, decreasing GHG emissions in the atmosphere through mitigation, adaptation, and resilience is a priority to avoid irreversible effects on the planet (Guemene Dountio et al. 2016).

The Paris Agreement emerged from a need to address this issue. It was signed by 195 countries in 2015 with the goal of limiting the global average temperature increase to 2 °C at a maximum (United Nations 2015). Colombia has engaged in reducing GHG and has established several circular economy strategies that allow its economic model the transition towards a greener economy, some examples are renewable energy diffusion, industrial symbiosis by “BORSI” program, implementation of the Circular Economic Strategy as public politic (first country in Latin-American in adopt it) (Álvarez-Espinosa et al. 2017; IPCC 2021; UNDP 2019), and implementation of a carbon tax. Colombia’s revised Nationally Determined Contribution (NDC) aims to reduce greenhouse gases by 51% and black carbon emissions by 40% by 2030 compared to 2014 levels (Gobierno de Colombia 2020). The black carbon target ensures Colombia’s NDC will improve air quality in Colombian cities, with significant health benefits, alongside climate mitigation.

Evaluating GHG emissions in educational institutes is the first step towards the proposed reduction targets at the local, national, and international levels. Several studies have reported the carbon footprint (CF) for university campuses under a life cycle assessment approach, aiming to reduce GHG emissions (Clabeaux et al. 2020). These reports also allow to strengthen plans and programs currently being developed in environmental management and recognize HEIs as entities that generate atmospheric emissions. For this reason, they must reduce their CF, comply with regulations, and contribute to improving environmental quality.

In this paper, we calculate the carbon footprint of the main urban campuses of Universidad Nacional de Colombia, Medellín, which recently has declared the climate crisis as a priority for our actions (UNAL Agencia 2021). For this analysis, we used the UNE-ISO 14064–1 and WRI/WBCSD GHG Protocol Corporate standard and the specific objectives are (i) to identify the different sources of direct and indirect greenhouse gas emissions generated by the university; (ii) calculate the carbon footprint, using the scopes established in the 2018 UNE-ISO 14064–1 standard as a reference; and (iii) define proposals and strategies for mitigating and reducing emissions. The main contribution of this work is to estimate the emissions of public HEI’s following a standard methodology. These results will provide a baseline for monitoring, evaluating, and establishing objectives for carbon management plans to reduce greenhouse gas emissions at points with the highest generation. Previous work (Aponte 2017; Barragan 2014; Manso et al. 2017; Reyes Salazar and Panche Cano 2019; Rojas and Chacón, 2011; Universidad Sergio Arboleda 2018) do not provide a clear analysis of the CF of Colombia HEIs due to the lack of use of the well-established and scientific-grounded methodologies of calculations, and high inventory data uncertainty. Regarding literature reviews on CF of international HEIs, results cannot be extrapolated to the Colombian case for the differences in the local conditions such as meteorological and whether, socioecological, policy and geographic conditions, lifestyle, university systems operations, amount others. This research can be a benchmark in the carbon footprint calculation and a methodological contribution to HEIs in the country.

The paper is organized as follows: in “The carbon footprint (CF) of HEIs: case studies,” a comparative analysis of the carbon footprint (CF) of different HEIs is performed to better understand their major GHG emissions and provide reduction strategies. “Materials and methods” presents the description of the case study and the detailed methodology used for the calculation. “Results and discussion” tackles the CF results, breakdowned by scope, source of emission, carbon footprint per capita, and type of greenhouse gas emission. The results are discussed and conclude that this study is a motivation to formulate and implement carbon sequestration strategies, which are being studied. Future research can use these results to suggest new policies for a sustainable campus.

## The carbon footprint (CF) of HEIs: case studies

Organizations contribute significantly to GHG emissions (Robinson et al. 2018), of particular importance are the HEIs because of the population of the university community, its physical size and infrastructure, and the complex combination of activities, such as education, laboratories, catering, retail, medical, and recreational facilities (Gu et al. 2019). It is estimated that there are more than 17,000 HEIs worldwide (Altbach et al. 2009), and the number of students attending university has grown exponentially since 2000 (Goddard 2011), especially in developing countries with more prominent environmental problems (Gu et al. 2018). The HEIs are key components of education systems worldwide, as they transcend international borders, socio-political regimes, and economic systems (Robinson et al. 2018). Also, HEIs are highly responsible for the production, continuation, and dissemination of knowledge (Otara 2014), and therefore, they play an important role in increasing awareness for contributing to sustainable development goals (Tan et al. 2014; Velazquez et al. 2006). CFs have been used for programs to mitigate climate change (Ridhosari and Rahman 2020), mainly, in those critical activities by implementing eco-efficiency and circular economy strategies that facilitate environmental, social, and economic decision-making processes. In particular, CF enable different organizations to:

(i) Identify hotspots for high-emission activities (Minx et al. 2009)
(ii) Streamline supply chains (Sundarakani et al. 2010)
(iii) Develop legitimate low-carbon products (Scipioni et al. 2012)
(iv) Define and prioritize climate policies for HEIs operation

Comparisons among CF in universities campuses studies are difficult given the heterogeneity across HEIs, in terms of population sizes, sources of GHG emissions, and variations in their carbon footprint methodology, particularly regarding scope 3 emissions. Some university campuses have higher CO_2 eq_ contribution from scope 3 to scope 2, followed by scope 1. However, the contribution from scope 3 is seldom the priority in carbon management policies due to is not mandatory intro the carbon footprint standardization method (Ozawa-Meida et al. 2011). The scope 1 emissions from owned or controlled emission sources or to the specific emission sources related to space heating or modes of transport. Some studies have chosen only activities, areas, or consumed or generated resources, regardless of the scope, as Pertamina University (Ridhosari and Rahman 2020), that is to say, does not break down the emissions by scope. In this work, we show the importance of reporting the CF by scope, as we developed Table 1, which allows a cross-comparison across HEIs.

**Table 1:** Report of CF emissions in different HEIs worldwide

*University/institution*	*Country*	*Year*	*Emission (t CO*_*2 eq*_*)*		*Scope*			
			*Total*	*Per capita* TM CO_2 eq_/person	*SP1* *%*	*SP2* *%*	*SP3* *%*	Reference
University of Leuven	Belgium	2010	7085	0.35	13.5	11.5	75	Lambrechts and Van Liedekerke (2014)
Clemson University	USA	2013–2014	95,418	4.3	19	40.6	40.4	Clabeaux et al. (2020)
Universitas Pertamina	Indonesia	2018–2019	1351.98	0.52	––-	98.96	1.04	Ridhosari and Rahman (2020)
Keele University	UK	2015–2016	14,272	1.3	46.7	41.5	11.8	Gu et al. (2018)
Autonomous Metropolitan University (UAM)	Mexico	2016	3000	1.07	4	24	72	Mendoza-Flores et al. (2019)
Bournemouth University	UK	2018	2119.6	1.43	10	31	59	Filimonau et al. (2021)
		2019	2139.6	1.41	9	27	64	
University of Medellin	Colombia	2016	1624	––	––-	––	––	^1^
Saint Thomas University	Colombia	2018	2415.8	0.069	18	34	48	Sebastián and Parra (2019)
University of Santiago de Compostela	Spain	2007	32,407.8	1.01	33	30.6	36.4	Hermosilla (2014)
De Montfort University	UK	2008–2009	51,080	1.99	6	15	79	Ozawa-Meida et al. (2013)
University of Valencia	Spain	2010	58,517.8	0.88	6.3	20	73.6	Hermosilla (2014)
National Autonomous University of Mexico, Engineering Institute	Mexico	2010	1577	1.47	5	42	53	Güereca et al. (2013)
University of Madrid School of Forestry Engineering	Spain	2010	2147	1.87	8.3	32.7	59	Alvarez et al. (2014)
Pontifical Catholic University of Rio de Janeiro, Gavea Campus	Brazil	2011	5782	0.29	1.5	0.2	98.3	de Carvalho et al. (2017)
University of Talca, Curico Campus	Chile	2012	1568,6	1	16	16	68	Vásquez et al. (2015)
University of Alberta	Canada	2012–2013	32,5351	6.51	52	40	8	Hyshka (2014)
Polytechnic University of Cartagena	Spain	2013	9008.4	1.07	3.6	16.9	79.4	Hermosilla (2014)
University of Valladolid	Spain	2014	22,080.5	1.1	24.6	30.2	45.2	Hernandéz and Cano (2014)
Edith Cowan University	Australia	2015	24,797.6	1.73	4	69	27	Favacho (2016)
University of Cambridge	UK	2016	102,049.9	3.5	20	52	28	Cambridge (2017)
University of California, Berkeley	USA	2016	151,650	2.9	44.2	28.1	27.7	California-Berkeley (2016)
University of Malaga	Spain	2017	24,831.6	0.66	2	57	41	Malaga (2017)
Autonomous Metropolitan University, Cuajimalpa Campus	Mexico	2016	2956,.3	1.07	4	24	72	Mendoza-Flores et al. (2019)
National Autonomous University of Mexico, Engineering Institute	Mexico	2010	1577	2.7	–-	–-	–-	Güereca et al. (2013)
Tongji University	China	2009–2010	NA	3.8	–-	–-	–-	Li et al. (2015)
The University of Cape Town, Africa	Cape Town	2007	84,926	4.0	–-	–-	–-	Letete et al. (2011)
University of Illinois at Chicago	USA	2008	275,000	10.9	–-	–-	–-	Klein-Banai et al. (2010)
University of Sydney	Australia	2008	20,000	–-	–-	–-	–-	Baboulet and Lenzen (2010)
University of Maribor	Slovenia	–	974	–-	–-	–-	–-	Lukman et al. (2009)
De Montfort University	England	2008–2009	51,080	2.4	–-	–-	–-	Ozawa-Meida et al. (2013)
Rowan University	USA	2007	38,000	4.0	–-	–-	–-	Riddell et al., (2009)
Clemson University	USA	2014–2017	95,418	4.4	–-	–-	–-	Clabeaux et al. (2020)
University of Castilla-La Mancha	Spain	2013	23,000	2.13	–-	–-	–-	Gómez et al. (2016)
Yale University	USA	2003–008	874,000	–-	–-	–-	–-	Thurston and Eckelman (2011)
Norwegian University of Technology & Science	Norway	2009	92,000	4.6	–-	–-	–-	Larsen et al. (2013)
University of Leeds	England	2010–2001	161,819	5.3	–-	–-	–-	Townsend and Barrett (2015)

CF estimation has been implemented in HEIs worldwide for 2 decades ago and most of them in the last decade as can see in Table 1. The HEIs have started to perform this accounting to optimize the resource utilization and to take environmental decision on the GHG emissions reductions. From Table 1, shown different carbon footprint of HEIs. For example, Clemson University’s GHG emissions are 19% for scope 1 and 41% for scopes 2 and 3, respectively (Clabeaux et al. 2020). The energy-water-carbon emission nexus analysis was evaluated at Keele University, policy suggestions are provided including implementing energy control systems, maximizing the development of wind energy and solar photovoltaic, increasing the availability of vegetable-based options in food procurement decisions, and collecting all of the food waste for anaerobic digestion (Gu et al. 2019). Keele University predominantly monitors carbon emissions from natural gas (scope 1) and electricity consumption (scope 2) (Gu et al. 2018).

Other carbon footprint results are University of Alberta in Canada (scope 1: 52%, scope 2: 40%, and scope 3: 8%) (Alberta 2014), California (Berkeley) (scope 1: 44.2%, scope 2: 28.1%, and scope 3: 27.7%) (California-Berkeley 2016), and Autonomous Metropolitan University (UAM) in Mexico City (scope 1: 4%, scope 2: 24%, and scope 3: 72%) (Mendoza-Flores et al. 2019). According to the consolidated information in Table 1, it is observed that the per capita emissions of some HEI register low values of 0.069 t CO_2 eq_/person and high values of up to 10.9 t CO_2 eq_/person, with average values of generation emissions of 2.28 t CO_2 eq_/person and a standard deviation of 2.22 t CO_2 eq_/person. This last result indicates the high degree of variability of the results and, therefore, their dependence on particularities that must be taken into account when performing the analyses.

On the other hand is the carbon footprint of Shikshana Prasarak Mandali’s Sir Parashurambhau, located in Western India, where scope 1, scope 2, and scope 3 contributed 28%, 48%, and 25%, respectively, to total emission. In this case, electricity, biodegradable and non-biodegradable waste, laboratory chemicals, paper, LPG, and transportation were the main contributors: Wageningen in the Netherlands (scope 1: 55%, scope 2: 20%, and scope 3: 25%) (University 2018), Cornell (scope 1: 76.4%, scope 2: 22.2%, and scope 3: 1.4%) (Cornell University 2018), Colgate in the USA (scope 1: 63.2%, scope 2: 3.7%, and scope 3: 33.1%) (Colgate University 2019). Bekaroo et al. in 2019 did not calculate the CF of university campuses. Instead, they researched personal CF contributions in HEIs (Bekaroo et al. 2019). Finally, typical research in a UK University calculated the CF during the COVID-19 lockdown. The main conclusion was that fully closing university campuses does not result in low GHG emissions (CF decreased by almost 30% during the lockdown). This is because the carbon benefits of online education are less significant than anticipated (Filimonau et al. 2021). Regarding the scope, the contribution was 6%, 21%, and 73% for scope 1, scope 2, and remote work/study, respectively.

Summarizing, the findings reported in Table 1 serve as a reference for policymakers and practitioners making decisions on the basis of sustainability in universities and other communities.

In the Colombian context, the majority of the CF reports from HEIs non-use of the rigorous data inventory or the non-implementation of the methodology principles: transparency, accuracy, consistency, full coverage, and relevance (NTC-ISO 2006). Consequently, it is implementing environmentally unfriendly strategies in the name of environmental protection to achieve competitiveness compared to other HEIs.

Similar to international studies, the CF assessment in Colombian HEIs has been performed with different details, scope, aims, and estimation methods (Varón-Hoyos et al. 2021); however, the majority of the reports have been used as undergraduate work and internal communication reports but not as scientific research.^1^ Some examples are the Sergio Arboleda University (Universidad Sergio Arboleda 2018), Jorge Tadeo Lozano University of Bogotá (Manso et al. 2017), Nueva Granada Military University (Barragan 2014), University of La Salle (Reyes Salazar and Panche Cano 2019), Industrial University of Santander (Rojas and Chacón 2011), University of Applied and Environmental Sciences (Aponte 2017), and University of Medellin.^2^ University of Medellin reports its emissions according to activities, with transportation (52%) and electric energy consumption (43%) as the most representative. One of the most recent CF research studies in a university campus in Colombia explains how scope 3 includes 98% of total GHGs (Varón-Hoyos et al. 2021). Colombian HEIs exhibit the lowest ton of CO_2 eq_. per person compared to HEIs in the rest of the world, because of a number of reasons, such as the low use of heating/cooling given the local climate conditions, and the lack of dormitories for international students.

## Materials and methods

### Study area—Universidad Nacional de Colombia (UNAL)

Universidad Nacional de Colombia (UNAL) is the largest public HEI in Colombia, with nine campuses throughout the country. UNAL Medellín is the second-largest UNAL campus, divided into eight main areas: three are main urban campuses, where it carries out most academic activities, a primary school, three agricultural stations, and one forestry station. For this research, the three main urban campuses were considered: El Volador, El Río, and Robledo. In Table 2, general information of the national university of Colombia headquarters Medellin is presented.

**Table 2:** General information of the National University of Colombia

Item	Value	Observations
Campus number	3	These campuses largely focus on carrying out the university’s mission, such as training competent and socially responsible professionals
Area	405,700 m^2^	
Students	12,610	Under and post-graduate
Academic programs	116	
Laboratories	142	
Professors	822	
Administrative employer’s	555	
Technical and professional service contractors	923	
Cafeterias^1^	16	
Buildings	58	
Sports venues	36,000 m^2^	
Green areas	260,934 m^2^	
Biological collection	1	The institution has and of, such as the “*León Morales Soto”* Arboretum and Palmetum, which is a biological collection with over 522 living species of trees and palms^2^

(UNAL 2019). (Facultad de Minas 2019)

^1^Universidad Nacional de Colombia – Noticias. URL: //medellin.unal.edu.co/noticias/514-la-coleccion-de-arboles-y-palmas-de-la-u-n-fue-designada-como-arboretum-y-palmetum-leon-morales-soto.html

^2^Arboretum and Palmetum at the Universidad Nacional de Colombia, Medellín. First Edition – December (2011)

### Carbon footprint methodology

There are several methodologies to quantify CFs, such as the PAS:2050 (Carbon Trust et al. 2008), BSI British Standard (BSI 2015), Green House Gas Protocol (Fong et al. 2012), Green Metrics (Universitas Indonesia 2010), and UNE-ISO 14064–1 (ISO 2019). All these methods suffer a common limitation of over or underestimation of GHG emission; for example, the GHG emissions for scope 3 are not standardized and depend on the availability of the data inventory or the research´s aim. The UNE-ISO 14064–1 and WRI/WBCSD GHG Protocol Corporate methodology was chosen to calculate the CF because it was created by a standard organization (ISO) and adopted by Colombia as NTC-ISO 14064–1. It is standardized, technically validated, and can be applied to any organization regardless of its economic activity, becoming a benchmark in terms of international standards. The IPCC implements this method to formulate public policies around the world. In addition, the United Nations Development Program evaluates the fulfillment of some SDG targets under this methodology (IPCC 2021; Sachs et al. 2021).

We have implemented the methodology through the following phases, according ISO 14064–1:2006. According to the literature review, this is the most used methodology in HEIs. The phases were (i) the organization’s limits, which included facilities the organization (voluntarily^3^) involved in the scope of CF calculating (working boundaries); (ii) data collection; (iii) the identification of sources of sources of emission for scope 1—direct emissions (sources owned or controlled by the organization), scope 2—indirect emissions (from generating heat, steam, or electricity from an external origin), and scope 3—other indirect emissions than those already included in scope 2 (modes of transport for students and employees, waste management, travels); and (iv) calculations and reports (NTC-ISO 2006).

The boundaries for calculating the CF of UNAL Medellín in this study include the administrative and academic activities performed in the three urban campuses (El Volador, Robledo, and El Río) by employees (administrative staff and professors), contractors, and visitors. The research included foodservice and food consumption in cafeterias and restaurants. The agricultural stations, forestry station, and the primary school were not included in the calculation of the CF, due to the fact that information on activities and values was not available. A reference year of this research is 2019.

### Carbon footprint data inventory of UNAL Medellín

Primary data was locally collected and categorized according to scopes 1, 2, and 3. Table 3 lists the data obtained for the Office of Information and Communications Technology, Transportation Section, Inventory Office, Environmental Management Office of Robledo Campus, El Volador Campus, and El Rio Campus.

**Table 3:** Carbon footprint data inventory of UNAL Medellín

Scope	Source		Activity	Value	Units
Scope 1	Gaseous fuel	Propane gas	Used in cafeterias	426	m^3^
		LPG	Used in boilers of dairy and wood laboratories	5146	m^3^
	Liquid fuel	Gasoline	Used for UNAL Medellín´s vehicles	33.24	m^3^
		Diesel		35.82	m^3^
Scope 2		Electrical network supply	Electricity consumption in physical units (e.g., offices, buildings, classroom)	5072.03	MWh
Scope 3	Transportation	Vehicle	Transportation by the university community to travel between the institution and their places of residence	20,805,450	km
		Motorcycle		6,523,920	km
		Bus		171,790	Passengers
		Subway		20	km
	Waste treatment/valorization /landfills	Incinerated waste	Hazardous waste produced at laboratories and other areas	5.08	t
		Post-consumer waste	WEEE, lamps, toner, used oils	1.71	t
		Deactivation	Biological waste, Chemical wastes	1.74	t
		Depressurization	Pressurized container waste	0.09	t
		Recycled waste	Recovered usable materials (paper, cardboard, glass, metals, etc.)	150.73	t
		Composting	Using organic waste (green waste and waste from cafeterias and/or restaurants. Composting is used for self-consumption	85.02	t
		Landfill	Ordinary and inert waste	134.16	t
		Wastewater	Dumping domestic wastewater into the sewage system	140,166	m^3^
	Internet network	E-mails	E-mails sent	1,8020,050	e-mails

#### Scope 1

This scope includes liquid fuel (gasoline and diesel) used by the institution’s vehicles (buses and cars) to transport students, teachers, and employees on routes between the campuses, agricultural stations, or other journeys. Gasoline consumption in the reference year is estimated to be 33.24 m^3^, while it was 35.82 m^3^ for diesel (Table 1).

Cafeterias and restaurants also consumed 426 m^3^ of propane gas to prepare and sell food for the university community. Finally, some teaching, research, and extension laboratories have boilers for dairy production and wood processing, which consumed about 5146 m^3^ of LPG.

#### Scope 2

The electrical network supply was obtained from the grid operator’s bills. The electrical network supply was obtained from the grid operator’s bills, which includes consumption (in MWh) for each campus. This consumption is for all the university’s urban campuses. Therefore, obtaining data discriminated by dependencies or buildings was not possible.

#### Scope 3

In this scope, we estimated the distances traveled by the university community from their places of residence to the university, and vice versa. The following considerations were made, according to the report on the Sustainable Mobility Plan of UNAL Medellín^4^:

- Vehicle: 3383 cars enter the university daily, with an average travel time of 30 min per trip. The average speed is 30 km/h. It is assumed there are two trips per vehicle (round trip) every day and 205 work days.
- Motorcycles: 936 motorcycles enter the university daily, traveling an average of 25 min each way. Their average speed is 40 km/h. We assumed motorcycle users made two trips per vehicle (round trip) per day, with 205 work days.
- Bus: 15% of the university community use the bus and spend an average of 40 min of transportation per trip. The average speed is 25 km/h. Therefore, we assumed 5% of a bus’ emissions are attributable to the University for Personnel Transportation, with 205 work days.
- Subway: 5% of the university community uses the metro, with 40 min per journey. We considered both ways to include a return trip. The commercial speed of the metro is 40 km/h.

Hazardous waste is normally generated in research laboratories. Chemical and biological waste is collected and treated (incinerated, deactivated, or depressurized) by an external manager with environmental permits from the competent authority due to their dangerous characteristics. During the reference year, there were 5.08 t of this waste. Biohazard waste generated in healthcare services within the campus corresponded to 1.74 t. It was deactivated through moist heat or chemical neutralization. Depressurized containers corresponded to 0.9 tons. Post-consumer waste (WEEE, used oils, lamps, and toners) was about 1.74 t, which were returned to producers through the Ministry of the Environment’s post-consumer programs for its recovery and treatment.

Recyclable waste corresponded to what was collected internally by the company RECIMED (150.73 t). Ordinary and inert waste (134,16 t) is taken to landfills by the public cleaning service company (EEVVM), and organic waste from cafeterias and restaurants is composted in the campus. In addition, aerobic composting is carried out with 185.02 t of waste. Domestic wastewater is approximately 140,166 m^3^. It is discharged through the sewage networks to be treated by the city’s public utility company.

Our objective in the research is to recognize the CF that is generated during the use and consumption of digital activities by the university community, which implies large energy and water consumption due to the use of servers for the operation of online services. Part of this footprint is included in scope 2 with the consideration of the electrical consumption of the institution’s internal servers. However, the university community also uses cloud services. For this reason, the number of sent e-mails works as a proxy to make this estimate more realistic. The number of sent e-mails by the university community is obtained from the Telecommunications Office of the institution. These emissions cannot be attributable to third parties since they are caused by members of the university institution. This study did not include saved e-mails and stored files, since they are stored on the university’s servers and their energy consumption is already accounted for in the energy bill. The number of e-mails sent by the university community is obtained from the Telecommunications Office of the institution. E-mails sent were 18,020,050 for the 2019 (Table 1). The number of e-mails sent is high since it is the main means of communication for the university community. In addition, the institution has had a zero-paper policy since mid-2016, which implies that a large part of the procedures was transferred to digital media. E-mails received were not counted because many of these are generated by and for the university community, causing double counting. The remaining e-mails received are assumed to be footprints produced by other institutions, entities, or people outside the institution. These emissions cannot be attributable to third parties since they are caused by members of the university institution.

### Carbon footprint calculation

CF is calculated for each scope according to carbon inventory data. For that, two steps must be performed, which are described below:

- GHG emissions (in tons of GHG) from activity data that produces the emission, through the following Eq. (1).

1 $$\mathrm{GHG\;emission}\left(\mathrm{t\;GHG}\right)=\mathrm{Activity\;data}\times \mathrm{Emission\;factor}$$

This equation applies to the following emissions:

- Combustion in fixed sources is usually expressed in energy units (TJ) and is calculated as the product of fuel consumption (in mass or volume) and the lower calorific value (PCI).
- Mobile combustion sources: activity data related to distance traveled (km) can be used. It can also be calculated based on the number of passengers transported.
- Electricity: the facility’s electrical consumption (expressed in kWh).

With respect to lower calorific value, Table 4 presents information about it for Colombian fuels. This information is necessary for direct fuel emission estimates and CO_2 eq_ emissions for scope 1.

**Table 4:** Lower calorific power of Colombian fuels

Fuels	Lower calorific power (MJ/kg o MJ/m^3^)	Source
LPG	45.4	UPME (2019a, b)
Gasoline	45.3	
Diesel	42.4	
Propane gas	46.2	

If the emissions are already given in terms of a specific GHG, this first step is issued, and the second is passed. Moreover, the emission factor is normally expressed in tons of GHG/unit and depends on the type and characteristics of the transformation process and type of fuel.

(b) Converting emission data from tons of GHG to tons of CO_2 eq_ through Eq. (2).

2 $$\mathrm{Emissions\;}\left(\mathrm{t\;}{\mathrm{CO}}_{2}\mathrm{equivalent}\right)=\mathrm{emission\;data\;}\times \mathrm{ potential\;global\;warming}$$

Global warming potential (at 100 years) is a factor that describes the impact of a unit’s radiation force based on the mass of a GHG to the equivalent unit of CO_2_ in 100 years. It is expressed in tons of CO_2 eq_/t GHG, and there is a factor for each type of GHG. Although The Intergovernmental Panel on Climate Change (IPCC) identifies that many gases have global warming potential (GWP) (Wiedmann and Minx 2008), there is not a unanimous consensus on the GHGs that should be included in a CF calculation. Therefore, this study only considered the six GHGs reported in the United Nations Framework Convention on Climate Change (UNFCCC) by the IPCC and its Kyoto Protocol (UNFCCC 2008), as shown in Table 5. Although Table 4 lists the most common greenhouse gases, only gases such as CH_4_, N_2_O, and CO_2_ were included in this study.

**Table 5:** Potential global warming

Greenhouse gas	Potential global warming	Source
CH_4_	25	IPCC – AR 4 – WG2 – Chapter 2
N_2_O	298	
CO_2_	1	
PFC_S_	9300	
HFC_S_	23,900	
SF_6_	22,800	

Once the unit calculation of the emissions from each source in units of tons of CO_2 eq_ was available, all emissions of the same category (direct emissions, indirect emissions for energy, and other indirect emissions) were added.

Finally, CO_2_ sequestration was estimated through Eq. (3). The absorption rate depends on each tree species. Sequestration will only be relevant when the organization has an agroforestry component or owns a significant area of land.

3 $${\mathrm{CO}}_{2}\mathrm{\;removal\;}\left({\mathrm{tCO}}_{2}\mathrm{equivalent}\right)=\mathrm{feet}\times \mathrm{ absorption\;rate}$$

where:

number of feet: the number of trees per species and per foot size (two kinds of trees are considered: those with larger feet, with a diameter greater than 5 cm, and smaller feet, with a diameter less than 5 cm). In addition, absorption rate is expressed in tons of CO_2 eq_/units per foot and year. Each species has an absorption rate.

For the case of the urban campuses of UNAL Medellín, this is a component to consider because UNAL-Medellín has extensive natural areas—about 260.934 km^2^. These areas are important because they generate multiple ecological services and are an important brick of the ecological structure of the city of Medellín and the Aburra Valley. The fact that these natural areas include the *Arboretum and Palmetum* collection, which hosts a sanctuary of fauna and flora inside the Metropolitan Area, is particularly relevant.

The *León Morales Soto Arboretum and Palmetum* collection is a botanical garden with approximately 445 species and about five thousand individual trees, palms, and bushes. It is a patch of urban forest matrix, making it one of the city’s green lungs. This collection provides multiple ecosystem services for the city, such as thermal regulation, pollution protection, wildlife habitat, CO_2_ sequestration, and water regulation.^5^

The forest inventory carried out in December 2016 was used to estimate GHG sequestration. In this inventory, 5351 individuals were registered in the three main campuses. For this study, it was impossible to consider the absorption factors reported by the IPCC because of the species listed, which almost entirely differed from those reported in the collection. For this reason, the average absorption factor was considered to be 0.02352 t of CO_2 eq_/year per individual, according to absorption rates raised by Ihobe (2006) in different regions of Asia.

With respect to the emission factors of Eq. 1, Table 6 lists the emission factors considered to estimate the CF.

**Table 6:** GHG emission factors

Source	Value	Units	Reference	Source	Value	Units	Reference
Scope 1				Scope 3			
LPG^2^	67,185.12	kg CO_2_/TJ	UPME, (2019a, b)	Vehicle^1^	172	gCO_2eq_/km	AMVA and UPB (2018)
	1	kg CH_4_/TJ		Motorcycle^3^	62.6	gCO_2eq_/km	AMVA and UPB (2018)
	0,1	kg N_2_O/TJ		Metro	28.27	gCO_2eq_/fare*km	Ríos et al., (2016)
Propane gas 1	55,539.09	kg CO_2_/TJ	IPCC (2006)	Bus^4^	291	gCO_2eq_/km	AMVA and UPB (2018)
	1	kg CH_4_/TJ					
	0.1	kg N_2_O/TJ		Landfill	587	kgCO_2eq_/tons	IPCC (2013)
Gasoline1	69,323.69	kg CO_2_/TJ	UPME, (2019a, b)	Incinerated waste	21.36	kgCO_2eq_/tons	IPCC (2013)
	3	kg CH_4_/TJ		Post-consumer waste	8.99	kgCO_2eq_/tons	IPCC (2013)
	0.6	kg N_2_O/TJ		Deactivation	21.36	kgCO_2eq_/tons	IPCC (2013)
Diesel1	74,193.48	kg CO_2_/TJ	UPME, (2019a, b)	Depressurization	99.76	kgCO_2eq_/tons	IPCC (2013)
	1	kg CH_4_/TJ		Recycled waste	8.28	kgCO_2eq_/tons	IPCC (2013)
	0.26	kg N_2_O/TJ		Composting	10.20	kgCO_2eq_/tons	IPCC (2013)
				Wastewater	73	kgCO_2eq_/person	IPCC (2007)
Scope 2							
Electricity1	0.199	kgCO_2eq_/kWh	UPME, (2019a, b)	E-mails	2.6	gCO_2eq_/ e-mails sent	**––-**

^1^The average value of the emission factor of vehicles measured for the Aburrá Valley of 1000cm^3^ and 1600cm^3^ is estimated because the type of vehicles that enter the institution daily has not been characterized

^2^The average value of the emission factor of motorcycles measured for the Aburrá Valley,at 100cm^3^, 110cm^3^, 125cm^3^, 150cm^3^, and 200cm^3^ is estimated because the motorcycles that enter the institution daily has not been characterized

^3^The average value of the emission factor of motorcycles measured for the Aburrá Valley,at 100cm^3^, 110cm^3^, 125cm^3^, 150cm^3^, and 200cm^3^ is estimated because the motorcycles that enter the institution daily has not been characterized

^4^The average value of the emission factor of diesel-powered vehicles with bus operation measured for the Aburrá Valley is estimated according to different cylinder capacities and models

## Results and discussion

This section presents the CF by scopes 1, 2, and 3, followed by the CF sequestration strategies. A comparative analysis is then approached, using UNAL-Medellin as a reference to other international HEIs.

### University campuses’ carbon footprint by scope.

After collecting the data for 2019, we estimated the CF considering the basis of calculation established in the previous section. The results are then discriminated by scope, source of emission, and type of GHG in some cases.

When it comes to scope 1, direct emissions from the liquid fuel consumption of the institution’s fleet of vehicles (mobile sources) and the consumption of gaseous fuels used in restaurants (fixed sources) operating in urban campuses generated a total of 418.63 tons of CO_2 eq_, as shown in Table 7. Liquid fuels represent the largest contribution, with 85.0%. These emissions are equal to 2.84% of the total percentage of emissions generated in UNAL. Of this percentage, 1.41% corresponds to mobile sources and the rest correspond to fixed sources.

**Table 7:** Quantification of direct emissions (scope 1) from UNAL urban campuses

Scope	Sources	Type of fuel	CO_2_ emissions (tons CO_2 eq_/yr)	CH_4_ emissions (tons CO_2 eq_/yr)	N_2_O Emissions (tons CO_2 eq_/yr)	Carbon footprint (ton s CO_2 eq_/yr)	% of scope 1	% of the total carbon footprint
**1**	Liquid fuel	Gasoline	77.23	0.0011	0.00011	77.30	37.86%	1.07%
		Diesel	95.90	0.0012	0.00031	96.29	47.17%	1.34%
	Gaseous fuel	LPG	15.70	0.00023	8.85734E-08	15.70	7.69%	0.22%
		Propane	14.86	0.000098	0.0000002	14.86	7.28%	0.21%
	SUBTOTAL		203.69	0.00263	0.0004	204.15	100.00%	2.84%

In terms of scope 2, the network operator in different contracts bills the energy consumption

Regarding scope 2, the network operator in different contracts invoices the energy consumption of the urban campuses of the UNAL. On a monthly basis, the electricity service operator measures consumption through devices and bills the university for the real value of electricity consumed. However, the value of annual consumption is totaled to calculate the carbon footprint. Therefore, a consumption of 5072.03 MWh is demonstrated for 2019, which translates into generating 1099.33 t of CO_2eq_, as seen in Table 8. This is equivalent to 14.03% of the total emissions generated by the institution. Although energy consumption is considerable, the CF does not represent a high contribution to the total CF because the Colombian energy matrix is one of the most renewable systems worldwide. The energy matrix is highly dependent on hydropower and natural gas, which represent 82% and 11%, respectively, of energy consumption in 2018 (UPME 2019b). On the other hand, thermal plant power generation has a participation of 6%, while contributions from other renewable energies do not exceed 0.1% (UPME 2019b).

**Table 8:** Quantification of indirect (scope 2) emissions from UNAL urban campuses

Scope	Source	Carbon footprint (tons CO_2 eq_/yr)	Unit	% of the total carbon footprint
2	Electrical network supply	1009.33	ton CO_2 eq_/yr	14.03%

Finally, sources of emissions not controlled by the entity are reported for scope 3. This scope includes (i) transportation by the university community to and from their places of residence, (ii) waste generation from the various activities carried out in the institution that is handled by third parties for its management and treatment, and (iii) sent e-mails. For this reason, generation is estimated at 5981.067 t of CO_2eq_, as presented in Table 9, which is equal to 83.13% of the total emissions generated by UNAL’s urban campuses and university community. Approximately 70% of these emissions come from transportation. Of this percentage, vehicle transportation was the main contributing factor. However, indirect emissions from transportation, such as commuting and university-related travel are more difficult to control, reason why additional projects are necessary for the university to reach carbon neutrality.

**Table 9:** Quantification of other indirect emissions from UNAL urban campuses (scope 3)

Scope	Sources	Type	Carbon footprint (tons CO_2 eq_/yr)	% of the scope	% of the total
3	Transportation	Vehicle	3.58	59.83%	49.74%
		Motorcycle	0.408	6.83%	5.68%
		Subway	0.097	1.62%	1.35%
		Bus	0.125	2.09%	1.74%
	Waste treatment/valorization /landfills	Landfill	78.75	1.32%	1.09%
		Incinerated waste	0.109	0.0018%	0.0015%
		Post-consumer waste	0.0154	0.0003%	0.0002%
		Deactivation	0.0372	0.0006%	0.0005%
		Depressurization	0.00898	0.0002%	0.0001%
		Recycled waste	1.25	0.021%	0.017%
		Composting	0.00087	0.00001%	0.00001%
		Wastewater	1223.772	20.46%	17.01%
	Internet network	E-mails	468.52	7.83%	6.51%
		Subtotal	5981.067	100.00%	83.13%

In summary, total GHG emissions in the urban campuses of UNAL-Medellín for each scope are listed in Table 10.

**Table 10:** Total emissions by scope on UNAL urban campuses

Scope	Subtotal (tons CO_2 eq_/yr)	% of the total
1	204.148	2.84%
2	1009.333	14.03%
3	6037.039	83.13%
Total	7250.52	100.00%

In addition, taking into account that there was a university population of 16,764 people during the study period, the institution’s per capita carbon footprint is estimated to be 0.432 t CO_2 eq_/person.

### Carbon sequestration

According to methodology described in the “Carbon footprint calculation,” it is estimated that CO_2_ sequestration by the institution’s biological collection is approximately 125.86 tons CO_2eq_/year and represents 1.74% of the greenhouse gases emitted in the institution.

### Comparative analysis of the carbon footprint of HEIs

UNAL Medellín is committed to reducing and mitigating GHG emissions. For this reason, it has calculated the institution’s CF in 2019. This study found that its CF was 7250.52 tons CO_2eq_, distributed as follows: scope 1 2.84%, scope 2 14.03%, and scope 3 83.13%. According to the Greenhouse Gas Protocol, the operational boundaries must consider scopes 1 and scope 2, while scope 3 is optional. Some elements from scope 3 were considered in this research. Consequently, the CF per person could have higher values than those of other international HEIs.

**Fig. 1:**
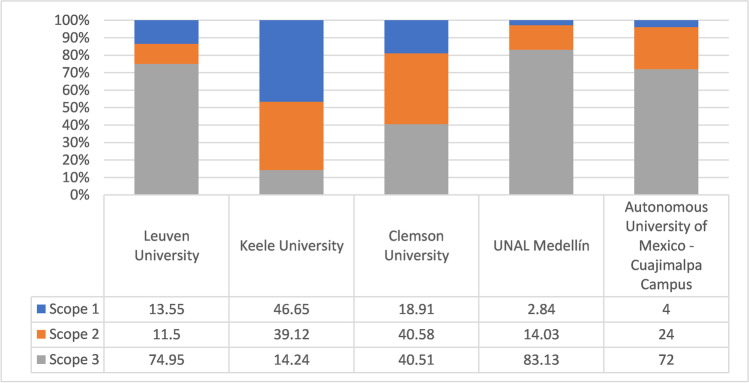
Comparison of the percentage distribution of UNAL Medellín’s scopes and those of other universities

Based in Table 1, Fig. 1 shows that the percentage distribution differs when comparing these values to those reported by institutions such as the University of Keele in the UK (Gu et al. 2019) and Clemson University in Southern California—United States (Clabeaux et al. 2020). The differences may be associated with the specific characteristics of each institution since the students of UNAL, as a public institution, have different economic conditions than students from private universities. These characteristics impact consumption factors, forms of food consumption, and transportation, leading to a lower contribution to the carbon footprint. Other causes that can affect the lower CF compared to other universities are on the university campus, heating is not required because there are not seasons in the country; the use of air conditioning is limited due to the high energy consumption generated by this equipment, which increases the electricity services payment; public HEIs could have less equipment for teaching and research therefore energy consumption is lower. In addition, it is necessary to consider that activities and sources were included in each scope, since this can mark the difference in calculations of total and per capita emissions.

For example, Clemson University (Clabeaux et al. 2020) estimated its CF at 95,418 tons CO_2_eq, where 19% corresponded to scope 1. This scope is one of the most represented in the CF, and 86% of the emissions accounted for in this range are associated with generating steam used for heating, domestic water, and dehumidification in the winter. This type of generation was also registered at the University of Keele (Gu et al. 2019) and the University of Leuven (Lambrechts and Van Liedekerke 2014) for the same purposes. However, this component was not observed at UNAL because of its geographical location. The above implies that the institution does not generate emissions associated with this activity. Therefore, values are lower in this scope.

When it comes to scope 2, the large emission gap associated with power generation can be related to generation sources (the energy matrix) in each country. For example, as Clabeaux et al. (2020) stated in their study, the generation matrix in California is mainly composed of 53% nuclear energy and 30% thermal energy. By contrast, Colombia has the sixth cleanest matrix globally, with 82% of installed capacity coming from renewable sources.^6^ This implies variations in the emission factor, which is greater for Clemson University. Therefore, this represents a greater share of emissions in the total CF estimate for that university.

**Fig. 2:**
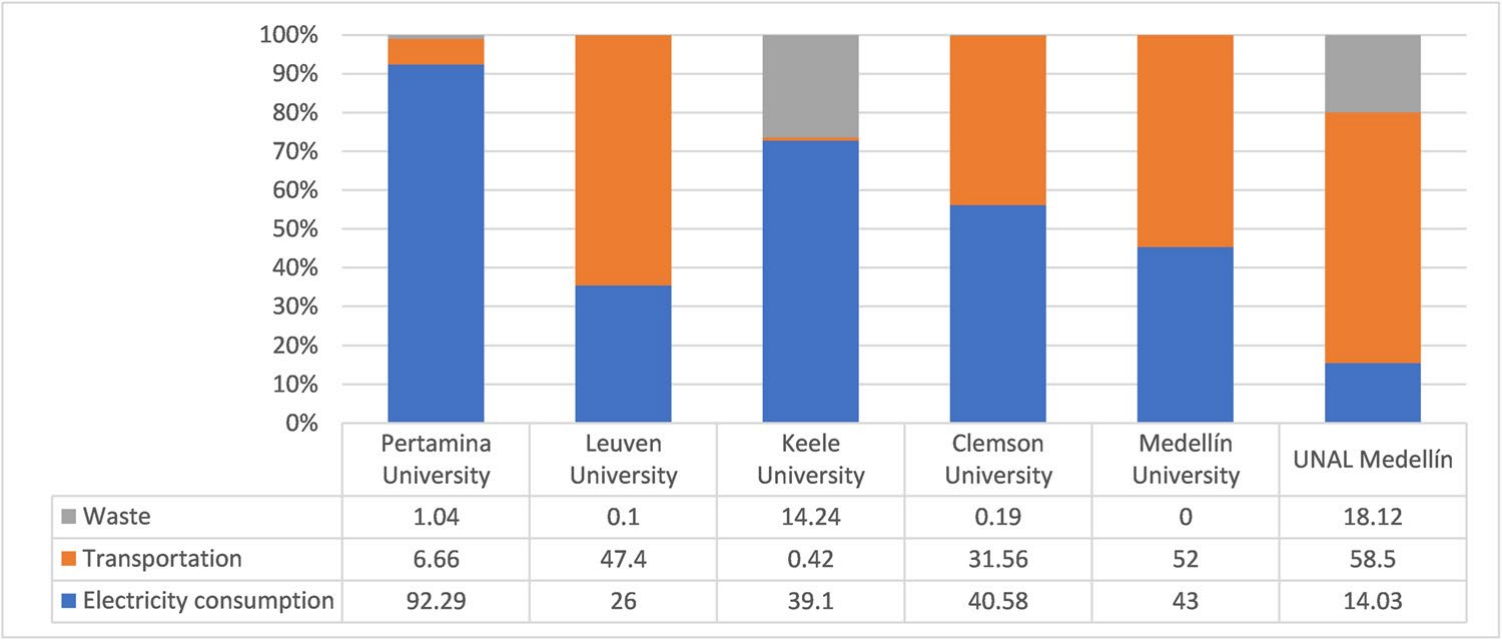
Comparison of the percentage of total emissions by components of UNAL Medellín and other HEIs

It is noteworthy that is not possible to compare the GHG emissions from activities linked to scope 3, due to the protocol lacks of the a standardization. UNAL Medellín had the greatest participation in scope 3 (83.13%), followed by the University of Leuven (74.95%). Although these values are relatively close, the sources and activities accounted for in this scope differ. For this reason, it is necessary to review the considerations made in each of the studies in detail, because it can demonstrate the differences in results regarding the rest of the universities. The above may be the case for the mobility/transportation and waste component. In Fig. 2, the results obtained by UNAL Medellín are compared with those of the universities mentioned above, in addition to Pertamina University in Jakarta-Indonesia (Ridhosari and Rahman 2020) and the University of Medellín^7^ in Colombia, according to the waste, mobility, and electricity consumption components.

The differences in the transportation component occur due to various reasons. The methodology used at the University of Pertamina (Ridhosari and Rahman 2020) included bicycles and public transportation, reducing the contribution of CO_2 eq_. The above may also be related to different geographical regions and the type of transportation. These conditions allow using private or public bicycles as more affordable means of transportation for all. This results in their carbon footprint being much smaller than UNAL’s for this component.

In the study conducted by Gu et al. (2019) for Keele University, only the contributions of emissions generated by the institution’s vehicles were considered. In contrast, Clemson University (Clabeaux et al. 2020) and University of Leuven (Lambrechts and Van Liedekerke 2014) included emissions generated by the university community with their vehicles and public transportation. Therefore, these sources were also included in this study. In the case of the University of Clemson, total emission values in this component were higher than UNAL Medellín’s because of the number of vehicles. For the first case, there were 16,521 vehicles, compared to 3383 in this study. The University of Medellín had values similar to those found for UNAL. The differences can be related to the population that manages each institution, type of academic offer (some programs involve several field trips), and fuel consumption for transportation to rural campuses.

The waste component had an 18.12% share in the CF of UNAL Medellín, with higher values compared to the University of Pertamina (1.04%), University of Leuven (0.1%), and Clemson University (0.19%). This estimate is related to the generation of hazardous waste that requires special management through incineration. This situation was not considered when calculating the waste component at the University of Pertamina (Ridhosari and Rahman 2020). In addition, in the research carried out by Lambrechts and Van Liedekerke (2014) for the University of Leuven, emissions generated due to wastewater treatment were not included. The University of Medellin does not record emissions associated with this component. It was found that the per capita CF is similar, between the range of 0.52 and 0.93 tons CO_2eq_. The University of Pertamina, University of Leuven, and UNAL Medellín had values of 0.52 tons CO_2 eq_, 0.93 tons CO_2 eq,_, and 0.432 tons CO_2_, respectively. Keele University had a CF of 4.4 tons CO_2 eq_, with the biggest difference between the mentioned institutions.

**Fig. 3:**
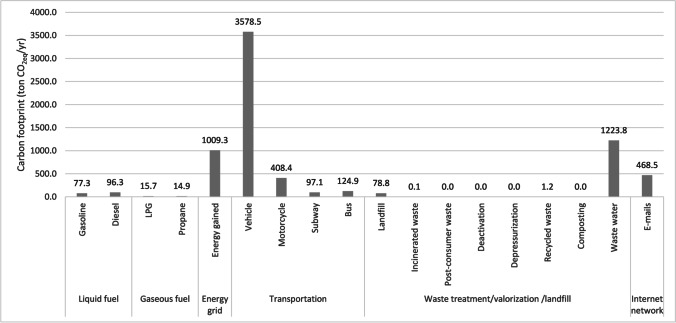
UNAL Medellín’s CO2eq emissions contribution analysis

Figure 3 shows the main sources that contribute to the CF by sources. The greatest contribution was from vehicle transportation (49.74%), followed by wastewater processes (17.01%), energy consumption (14.03%), and sent e-mails (6.51%). With that in mind, UNAL Medellín needs to implement actions to reduce and mitigate the large GHG emissions from these sources. The institution has implemented various strategies to incentivize changing transportation methods. Some of them included educational persuasion and teleworking. The university recently installed electric charging stations in the Robledo and El Volador Campuses for cars, motorcycles, and bicycles. Additionally, UNAL Medellín has implemented a photovoltaic energy system that generates approximately 112 MWh, providing 2.16% of the energy consumption from the Colombian electrical grid in 2020. This represents a 2.2% reduction in CO_2 eq_ emissions. Our institution expects to self-generate an additional 1140 MWh of energy by installing more panels in different buildings by the end of 2021. In addition, UNAL has worked on campaigns to reduce the digital CF by taking into account the use of computers, internet browsers, and sending e-mails.

Regarding the contribution of wastewater to the carbon footprint, it has already been mentioned that the institution does not have a domestic or industrial wastewater treatment plant. The wastewater generated in various activities is discharged through the sewage network to be treated at the municipal treatment plant later. However, the wastewater generated in laboratories is deactivated or neutralized before discharging it to reduce its potential danger. In terms of the consumption of water, which becomes domestic wastewater, some taps that regulate the flow have been changed to avoid the loss of water. In addition, the Environmental Management Office of UNAL Medellín will implement the water footprint methodology to calculate the direct and indirect green, blue, and gray water footprint and the sustainable water footprint index to implement optimization strategies. Educational campaigns have also been carried out for all university personnel to reduce water consumption and avoid waste. Similarly, obsolete sanitary batteries and those in bad conditions have been replaced to reduce water consumption and, therefore, wastewater generation. The institution has also carried out two planting days and sown a total of 150 individual trees. Some of the species planted include *Andira Inermis*, *Koelreuteria Bipinnata*, and *Swartzia Robiniifolia*, which are typical of the geographic location.

**Fig. 4:**
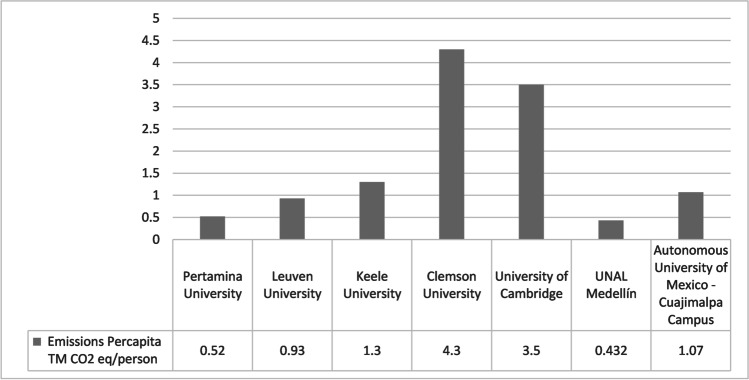
Comparison of CO_2_eq emissions per capita between UNAL Medellín and other universities

Finally, when comparing CO_2 eq_ emissions/person, Fig. 4 shows a large variability. Multiple reasons can be incorporated, such as the income conditioned by the GDP of each country, as it is the case of Clemson University and Cambridge University compared to Pertamina and UNAL Medellín. GHG emission sources (renewable and non-renewable resources, variability in the GHG emissions considered into scope 3), climate variability (use or not of calefaction/air conditioned), consumption patterns, composition of the energy matrix for each country, uncertainties from characterization factors, among the main.

We have also compared the CF between different HEIs, despite the limitations due to the dependence on multiple determining factors, such as geographic location (climate and topography), cultural factors (consumption patterns and types of transportation), population size, typology (public or private), and methodology selection. GDP plays an important role in the HEIs’ GHG emissions because more income means more consumption and more GHG emissions. However, comparisons between studies that have reported the CF of university campuses are difficult because the HEIs have ranging population sizes, GHG emission sources, and variations in their carbon footprint methodologies, particularly regarding scope three emissions. Uncertainties from characterization factors should be into account in future works due to could present change both the results and mitigation strategies.

## Conclusions

In this study, we estimate the UNAL University’s CF, which was 7250.52 tons CO_2 eq_, and 0.432 tons CO_2 eq_/person in 2019, where the 2018–2019 period is the baseline and the gas emissions reduction strategies to be implemented in the following years will be evaluated based on it. According to the Greenhouse Gas Protocol, the operational boundaries must consider scope 1 and scope 2, while scope 3 is optional. In this research, some elements from scope 3 were considered, for which reason the CF per person could be higher compared to other international HEIs. Scope 1 emissions accounted for about 2.84% of the carbon footprint, while scope 2 and 3 emissions each contributed nearly 14% and 83%, respectively. Electricity consumption, waste generation, and sent e-mails were the main contributors to the generations of GHG emissions, with contributions of 14.03%, 20.46%, and 7.83%, respectively. This work is particularly relevant considering that the University Council has declared as a priority all actions towards improving the climate crisis. We have compared the CF between different HEIs, despite the limitations due to the dependence on multiple determining factors. This estimation is crucial to formulate sustainable strategies, public policies, and an important step in transforming a Campus into a Sustainable, zero-CF Campus.

UNAL Medellín has a representative collection of tree species that capture 1.74% of CO_2 eq_. Nevertheless, it is evident that this percentage is low compared to the contribution of the GHG sources in the study. Therefore, planting more trees is not very effective if capturing emissions is analyzed in isolation. In this sense, generating awareness and alternative policies aimed at reducing greenhouse gas emissions is also required. This includes the conversion or transition from conventional to electric vehicles, efficient use of utilities (energy and water), use of alternative energy sources and rainwater, minimization of solid waste generation, use of bicycles, among others.

This study estimated total sequestration value based on an average value of emission factors from literature, adapted to local characteristics. Therefore, further research could study the contribution of the species located in the institution to offsetting GHG emissions. Additionally, it is recommended to include future CF information corresponding to agricultural research centers, since they contribute considerable CO_2 eq_ figures due to their livestock and agricultural activities. It is important to consider implementing carbon bond market into HEIs as verification by a third party that some organizations or individuals removed or avoided the emission of one ton of CO_2_e. This is because the Paris Agreement’s 2 °C scenario requires adopting high-efficiency and low-carbon systems.

Further work can be framed on the carbon footprint HEIs calculation patrons, which would allow getting a carbon footprint calculation framework in HEIs. As a consequence, the variability of the CF per capita and by scope can be minimized at least to the reasons that are inherent to the method implementation, scope, and parameters defined (calculus patrons).

## Footnotes

1 We do not consider the internal communication report as scientist research because these reports don’t have a rigorous methological, do not clear the data source and inventory, and finally, do not possible reply the results obtained.

2 Medellin University – Campus Vivo. URL: https://www.udem.edu.co/index.php/gestion-de-la-sostenibilidad/cambio-climatico-y-resiliencia-campus-vivo#:~:text=La%20Universidad%20de%20Medell%C3%ADn%2C%20lleva,con%20una%20reducci%C3%B3n%20del%2020%25.

3 The word “voluntary” in this sentence refers to the free or flexible character that organization must include or not spaces or facilities within CF estimate.

4 The Sustainable Mobility Plan aims to reduce the emission of polluting agents and greenhouse gases through cultural transformation by leading to reflection on the impact of their journeys. For its construction, it is necessary for the institution to register information on the modes of transportation used by the community, the number of vehicles that enter the institution, distance, and transportation time, among others.

5 Universidad Nacional de Colombia – Noticias. URL: //medellin.unal.edu.co/noticias/514-la-coleccion-de-arboles-y-palmas-de-la-u-n-fue-designada-como-arboretum-y-palmetum-leon-morales-soto.html.

6 Asociación Colombiana de Generadores de Energía Eléctrica -ACOLGEN-. URL: https://www.acolgen.org.co/.

7 Medellin University – Campus Vivo. URL: https://www.udem.edu.co/index.php/gestion-de-la-sostenibilidad/cambio-climatico-y-resiliencia-campus-vivo#:~:text=La%20Universidad%20de%20Medell%C3%ADn%2C%20lleva,con%20una%20reducci%C3%B3n%20del%2020%25.
